# COVID-19 Related Stress and Mental Health Outcomes 1 Year After the Peak of the Pandemic Outbreak in China: the Mediating Effect of Resilience and Social Support

**DOI:** 10.3389/fpsyt.2022.828379

**Published:** 2022-02-21

**Authors:** Jingchu Hu, Yiting Huang, Jiayu Liu, Zhiying Zheng, Xiuhua Xu, Yunfei Zhou, Jianhong Wang

**Affiliations:** ^1^Department of Anxiety Disorders, Shenzhen Clinical Research Center for Mental Illness, Shenzhen Kangning Hospital, Shenzhen, China; ^2^Shenzhen Clinical Research Center for Mental Illness, Shenzhen Kangning Hospital, Shenzhen, China

**Keywords:** COVID-19, stress, mental health, social support, resilience

## Abstract

**Background:**

COVID-19 outbreak have a long-term negative impact on mental health. Meanwhile, it may also provide opportunities for positive outcomes (e.g., post-traumatic growth). Resilience and social support could serve as psychological resources to protect individuals against the detrimental effects of the COVID-19 crisis and enable people to develop positive changes during challenging times.

**Objective:**

By testing the roles of resilience and social support in the relationship between COVID-19 related stress and negative mental health outcomes (depression and anxiety), as well as the relationship between COVID-19 related stress and positive mental health outcomes (post-traumatic growth, PTG), this study aimed to investigate the psychological mechanisms involved in different mental health outcomes induced by COVID-19.

**Methods:**

An online survey was conducted 1 year after the peak of the COVID-19 outbreak (from April to August 2021) in China. The survey includes demographic questionnaires and six scales: the Impact of Event Scale-Revised for COVID-19 (IES-RC), the 10-item Connor-Davidson Resilience Scale (CD-RISC-10), the Perceived Social Support Scale (PSSS), the Center for Epidemiological Studies Depression Scale (CES-D), the Generalized Anxiety Disorder scale (GAD-7) and the Posttraumatic Growth Inventory (PTGI). The structural equation model (SEM) was used to evaluate the relations and mechanisms between COVID-19 related stress and resilience, social support in depression, anxiety, and PTG.

**Results:**

A total of 771 Chinese subjects completed the questionnaire, including 416 (54%) females. COVID-19 related stress was associated with anxiety (*P* < 0.001), PTG (*P* < 0.001), and depression (*P* < 0.001). Resilience was related to depression (*P* < 0.001), anxiety (*P* < 0.001), and PTG (*P* < 0.001). Social support was associated with depression (*P* < 0.001), anxiety (*P* < 0.001), and PTG (*P* < 0.001). Under SEM analysis, resilience mediated the effects of COVID-19 related stress on depression and post-traumatic growth. Social support mediated the impacts of COVID-19 related stress on post-traumatic growth, depression, and anxiety. The path coefficients of the mediation effects were statistically significant.

**Conclusions:**

The current findings suggest that COVID-19 related stress has a double-edged effect on mental health. Depression, anxiety, and PTG coexist in Chinese individuals 1 year after the peak of the pandemic. Resilience and social support serve as important protective factors of mental health, safeguard people from the negative mental health outcomes of the COVID-19, and promote PTG.

## Introduction

**Figure 1:**
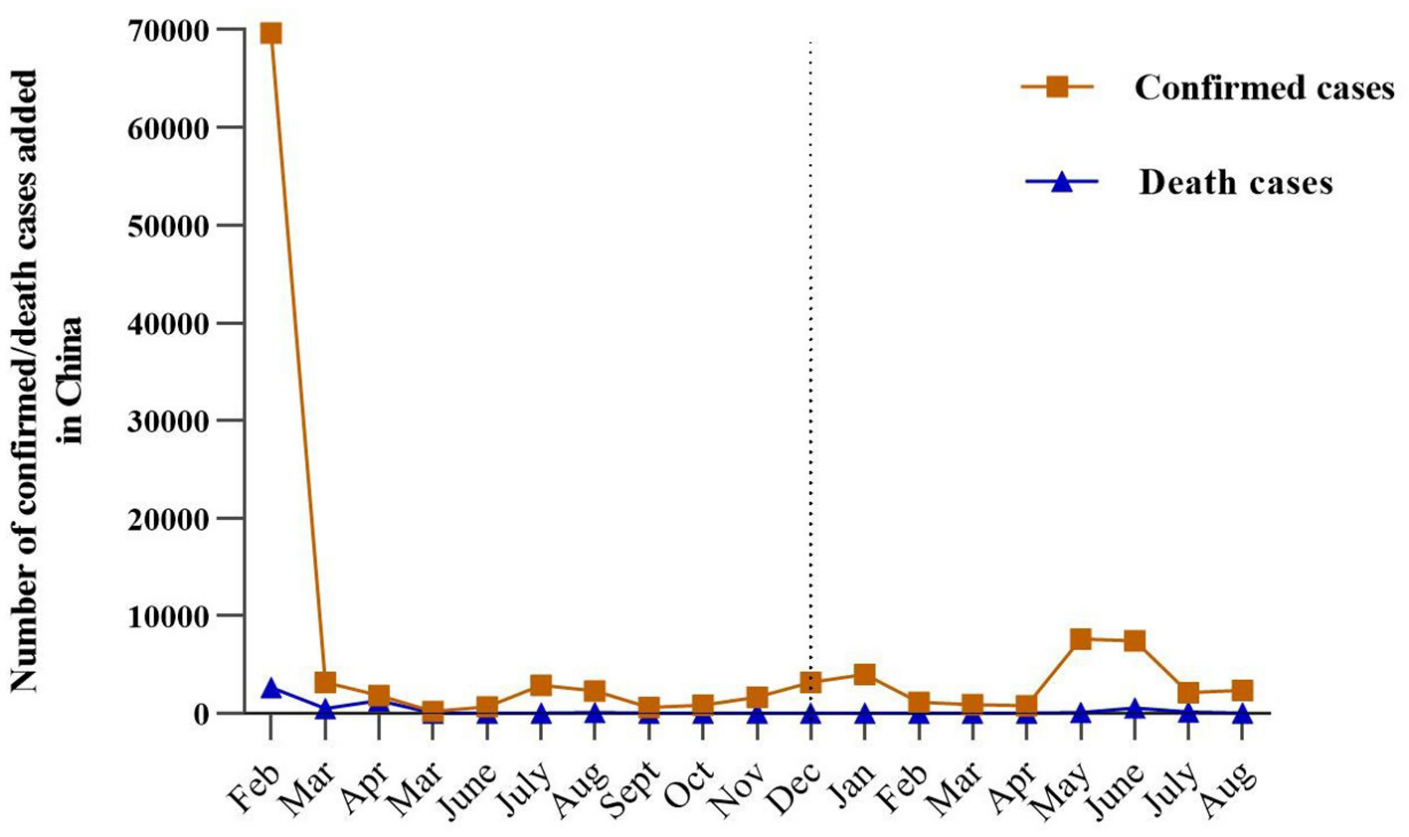
Trajectory of the COVID-19 pandemic in China (including Hong Kong, Macao, and Taiwan) with the number of confirmed and deaths cases added each month from February 2020 to April 2021. The left side of the dotted line is 2020, and the right side is 2021.

The Coronavirus-19 (COVID-19) pandemic began as viral pneumonia in China in December 2019 and has posed a severe threat to people's mental health globally with its lethal spread. The rapid development of the pandemic and the following restrictive quarantine measures (e.g., isolation at home) had a profoundly psychological impact on most people. A nationwide survey conducted at the peak of the pandemic in China reported that around 35% of the respondents experienced psychological distress (1). Another Chinese study found that 53.8% of the respondents had experienced psychological impacts of the COVID-19 pandemic on a moderate or severe level, with 8.1% of respondents reporting moderate to severe stress levels by early 2020 (2). These negative mental health outcomes may attribute to the stress induced by the COVID-19 pandemic, as some researchers indicated that COVID-19 related stress made people more vulnerable to developing mental health issues (3, 4). Recent studies further confirmed an association between COVID-19 related stress and negative mental health outcomes (5–7). Besides, some researchers found that COVID-19 related mental health outcomes are not static but dynamic events that fluctuate with the number of infected cases (8). Therefore, it is crucial to understand the mental health outcomes and the influencing factors in periods with different infected cases during the COVID-19 pandemic. In early 2021(one year after the peak of the pandemic in China), as a result of effective treatments and preventions made by the government, China continued to report a lower number of new coronaviruses confirmed cases (28 confirmed cases by 1 April 2021) and 0 new deaths (9, 10) (see Figure 1). However, few studies explored the impact of COVID-19 related stress on mental health outcomes after a sharp drop in infection cases and deaths.

Despite the mental health concerns of the COVID-19 pandemic attracting significant attention, recent researchers questioned whether COVID-19 acts as a stressful event and offers opportunities for people to grow (8, 11). Previous studies demonstrated that the phenomenon of post-traumatic growth happened after the SARS pandemic (12). Post-traumatic growth (PTG) was defined as positive change after experiencing the trauma in people's self and life (13). The development of PTG encouraged individuals to appreciate more about their life, improve self-perception, and develop intimate relationships with others after surviving from trauma (13, 14). A nationwide survey among 2038 Chinese university students reported that 66.9% experienced post-traumatic growth during the COVID-19 pandemic (15). Nevertheless, only a few studies have investigated post-traumatic growth and the potential mechanisms involved during the COVID-19 pandemic. None of the studies discussed post-traumatic growth caused by COVID-19 related stress and the associated psychosocial factors 1 year after the peak of pandemic.

Among all the possible influencing factors on the mental outcomes induced by the COVID-19 pandemic, resilience has been recognized as a significant influence factor (8, 16). Resilience refers to the cognitive process of adapting well in the face of adversity (17). The framework proposed by previous researchers suggested that resilience is the central part of the recovery from trauma or adversity (18). It can be considered as personal strength for individuals to protect their mental health and enable them to cope with traumatic events (19, 20). Recent studies indicated that a higher level of resilience in individuals predicted lower depression symptoms and anxiety symptoms during the COVID-19 pandemic (21–23). As a positive resource, other research also found that resilience could facilitate the development of PTG (16, 24). When people go through the adversity of COVID-19, resilience may work as a crucial factor in reducing the stressful experience, consequently maintaining people's mental health. In line with this, abundant research has demonstrated the indirect effect of resilience between stress and mental health outcomes such as depression and anxiety (7, 25).

In the meantime, the social-cognitive theory underlined the salience of social support in facilitating active cognitive processing and finding positive meaning (26). Social support is defined as the assert of effective social networks and supportive relationships with the therapeutic effects on mental health (27). It can serve as a buffer to the severity of the traumatic events and foster people's recovery from the difficulties (27). As a coping resource, empirical studies indicated that a higher level of support from family, friends, and significant others would predict a higher level of post-traumatic growth under the context of the COVID-19 pandemic (28–30). Meanwhile, perceived social support works as a protective factor in reducing depression and anxiety during the COVID-19 pandemic (31, 32). In addition to the direct effects, a recent study proved the indirect impact of social support in the association between perceived stress and depression (33). Moreover, social support is recognized as an important source of mental health for Chinese people under collectivist culture (34).

**Figure 2:**
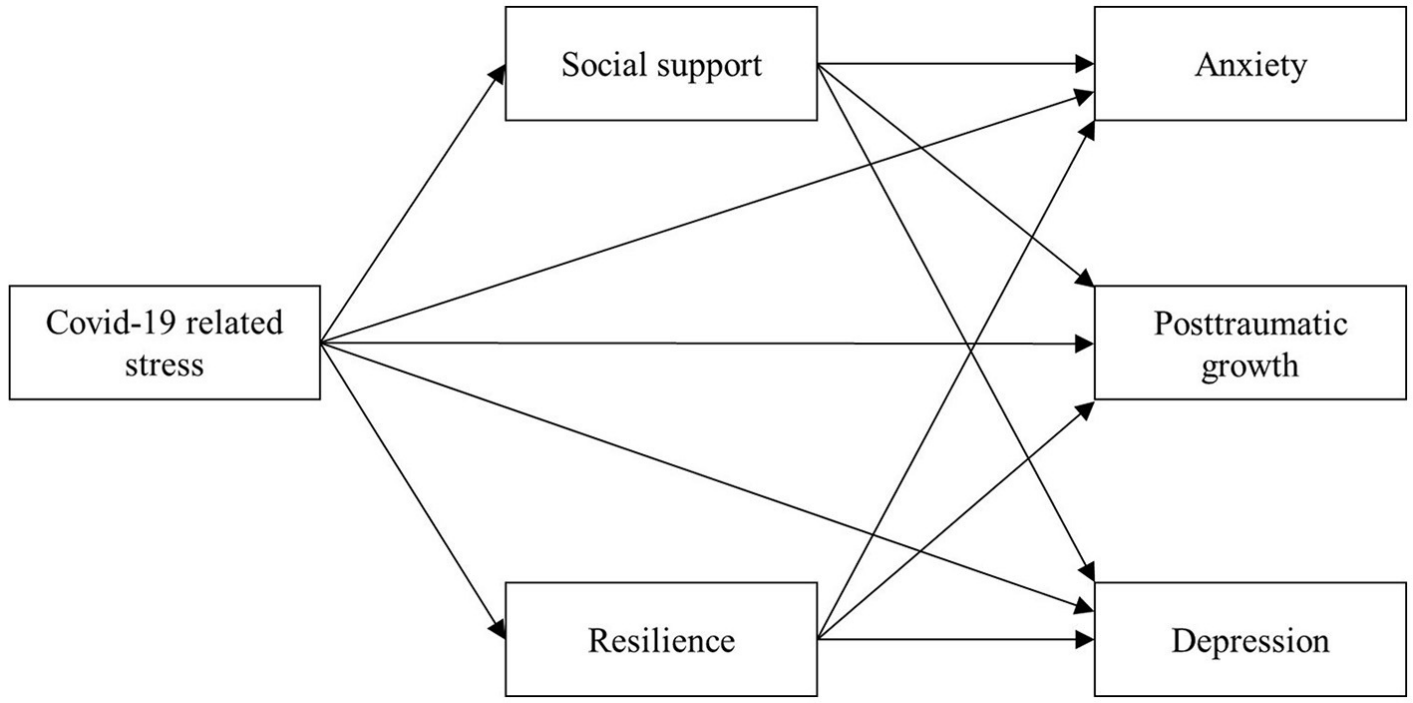
Hypothesized conceptual framework indicating the relationships among the COVID-19 related stress, social support, resilience, and mental health outcomes (depression, anxiety, and posttraumatic growth).

Although extensive research confirmed the negative impacts of COVID-19 related stress on individuals' mental health, it is still unclear whether there are any positive impacts of COVID-19 related stress and the potential mechanisms underlying them. Since China is the first country that experienced a sharp fall in the number of confirmed coronavirus cases for 1 year (see Figure 1), the research into mental outcomes affected by COVID-19 related stress among Chinese people could provide leads for further investigations in the process of stress-related growth in the new stage of the pandemic. Thus, the purpose of this study is to evaluate the indirect roles of resilience and social support between COVID-19 related stress and negative/positive mental health outcomes (depression, anxiety, and PTG) among the Chinese 1 year after the peak of the COVID-19 pandemic. The framework of the current study was proposed and shown in Figure 2. The study addressed the following hypotheses: (1) COVID-19 related stress is positively associated with depression, anxiety, and post-traumatic growth. (2) Resilience and social support are negatively associated with depression and anxiety but positively associated with post-traumatic growth. (3) Resilience and social support mediate the relationship between COVID-19 related stress and mental health outcomes (depression, anxiety, and post-traumatic growth).

## Methods

### Participants and Procedures

A cross-sectional online survey was conducted 1 year after the peak of the COVID-19 outbreak (from April to August 2021) in China. The present study was approved by the Institutional Review Board of Kangning Hospital (code: 2020-3-20-2). This study used the structural equation model (SEM) for the data analysis, and the previous research suggested the sample size of the structural equation model should be over 200 (35). A total of 771 participants was recruited, which included 54% female (for more specific demographic information, see Table 1). All of participants were provided informed consent before filling out the survey. Considering the spread of the epidemic, an online platform performed the data collection procedure, www.wjx.cn, a widely used survey distribution and data collection website in China. To ensure the data reliability, except all participants were anonymous, we set up validation questions in the questionnaire. Participants that included in following data analysis should meet (1) answered the validation questions correctly (e.g., What is the capital city of China?) (2) answered all the questions thoughtfully. Finally, all eligible participants were provided the same compensation.

**Table 1:** Participant demographic information (*N* = 771).

**Characteristics**	***n***	**%**
**Sex**		
Male	355	46.0
Female	416	54.0
**Whether in only-child family**		
Yes	285	37.0
No	486	63.0
**Age, y**		
<20	29	3.8
20–29	398	51.6
30–39	278	36.1
40–49	57	7.4
50–59	9	1.2
**Education**		
≤ Junior high school	7	0.9
Senior high school	46	6.0
College	122	15.8
Undergraduate	554	71.9
≥ Postgraduate	42	5.4
**Household income, yuan^#^**		
<50,000	54	7.0
50,000–100,000	190	24.6
100,000–200,000	309	40.1
200,000–500,000	188	24.4
500,000–1,000,000	27	3.5
>1,000,000	3	0.4
**Career**		
Worker	64	8.3
Former	5	0.6
Student	126	16.3
Medical staff	21	2.7
Educational, scientific and Cultural personnel	45	5.8
Enterprise manager	334	43.3
Government/public Institution personnel	84	10.9
Migrant worker	25	3.2
Other	67	8.7

# *1 yuan = 0.16 dollar, updated by January 6th, 2022*.

## Measurements

### COVID-19 Related Stress

The COVID-19 related stress was assessed by the Chinese version of the Impact of Event Scale-Revised Version (IES-R) (36, 37), a 22-item measure reaction adapted to the COVID-19 related events (Supplementary Table 1). According to the last seven days' stress level caused by the COVID-19, participants were required to rate on a five-point Likert scale from 0 (“not at all”) to four (“extremely”). The adapted items, for example, “I tried not to think about COVID-19”. This scale includes three dimensions: intrusion, avoidance, and hyperarousal. The Cronbach's α coefficient was 0.852 in the present sample.

### Generalized Anxiety Disorder Scale

Participants' anxiety symptoms were assessed by the Chinese version of the Generalized Anxiety Disorder (GAD-7) scale (38, 39). Based on the past 2 weeks' experience, participants rated how often they have been bothered by the seven anxiety symptoms from 0 (not at all) to three (nearly every day). The total score of this scale ranges from 0 to 21. The Cronbach's α coefficient for this measure was 0.842.

### Post-traumatic Growth Inventory

The Chinese version of the Posttraumatic Growth Inventory (PTGI) (14, 40) was used to measure post-traumatic growth. The scale consists of 21 items with five dimensions: relating to others, new possibilities, personal strength, spiritual change, and appreciation of life. Six-point Likert scale was used for this scale from “I did not experience this change as a result of my crisis” to “I experienced this change to a Very great degree as a result of my crisis”. The single item score ranged from 0 to five, and the total score ranged from 0 to 105. A higher score indicates a higher level of post-traumatic growth. The Cronbach's α coefficient for the PTG inventory was 0.944 in this study.

### Center for Epidemiological Studies Depression Scale

The original English version of the Center for Epidemiological Studies Depression Scale (CES-D) is a 20 items scale that measures the participant's depression symptoms during the last week, rating from 0 (rarely or none of the time) to three (most or all of the time) (41). The total score of this scale is 60. The participant's score higher than 15 implies clinically depressive symptoms (40). The Chinese version of CES-D that had been validated in previous studies was performed in this study (42, 43). For the present study, the Cronbach's α coefficient for the CES-D was 0.908.

### Multidimensional Scale of Perceived Social Support

The Chinese version of Multidimensional Scale of Perceived Social Support (MSPSS) (44, 45) is a 12-item self-reported measure and was used to measure the level of social support from three dimensions: family, friends, and others. The scale is a seven-point Likert scale from one (very strongly disagree) to seven (very strongly agree), the higher scores that participants rated indicated higher perceived social support. The Cronbach's α coefficient for this scale is 0.894.

### 10-Item Connor-Davidson Resilience Scale

The 10-item Connor-Davidson Resilience Scale (CD-RISC-10) is a 10 items scale measured in a five-point Likert scale from 0 (not true at all) to four (true nearly all the time) (46). The translated Chinese version was used in this study (47). The scale score ranges from 0 to 40, the higher score suggesting a better resilience capability. For the present sample, Cronbach's α coefficient was 0.846.

### Data Analysis

Data analyses were performed with IBM SPSS statistical version 23.0 (IBM Corp) and Mplus 8.3. Only completed questionnaires were included in the analysis, and there were no missing data. To examine the hypotheses, descriptive analysis, correlation analysis, and structural equation modeling were conducted, respectively. Demographic information, like gender and age, was provided by number (*n*) and percent (%). The continuous mental health variables, like the COVID-19 related stress and PTG, were provided by mean (*M*) and standard deviation (*S.D*.). Kolmogorov-Smirnov statistical test was run to examine the normality of data distribution. The results showed that data were not normal distribution. Therefore, the Spearman correlation analyses were carried out to explore the associations among the key variables. The statistical significance was set at *P* < 0.05, and all tests were two-tailed. As the data were not normal distribution, bootstrapping (with 5,000 re-samples) was adopted in Structural equation modeling to test the significances of relationships among the key variables by controlling all demographic variables (i.e., conceptual model), and especially we would like to explore how COVID-19 related stress may shape three mental health outcomes directly or indirectly by the social support and resilience variables as mediators. The following related indices were used to examine the final model fit (48): a non-significant chi-square (χ2), the root mean square error of approximation (RMSEA <0.08), the comparative fit index (CFI > 0.09), and the Tucker–Lewis index (TLI > 0.90). *Post hoc* power analysis indicated 771 participants showed a good fit the model with power close to one when RMSEA was between 0 and 0.08.

## Results

### Descriptive Statistics and Correlation Analysis

A total of 771 eligible participants were included in the final analysis. Table 1 presents demographic characteristics. Bivariate correlation analyses were performed to investigate possible associations among the key study variables. Means, standard deviations, and correlations of the key study variables are shown in Table 2. The result indicated that the COVID-19 related stress was positively related to anxiety [*r*_(769)_ = 0.609, *P* < 0.001], PTG [*r*_(769)_ = 0.213, *P* < 0.001], and depression [*r*_(769)_ = 0.497, *P* < 0.001], supporting hypothesis 1. Resilience was negatively associated with depression [*r*_(769)_ = −0.0386, *P* < 0.001] and anxiety [*r*_(769)_ = −0.297, *P* < 0.001], while positively associated with PTG [*r*_(769)_ = 0.395, *P* < 0.001]. We found similar results in social support, which was negatively associated with depression [*r*_(769)_ = −0.482, *P* < 0.001] and anxiety [*r*_(769)_ = −0.384, *P* < 0.001], but positively associated with PTG [*r*_(769)_ = 0.355, *P* < 0.001]. Besides, the results of the correlation provided insights for further investigation.

**Table 2:** Descriptive statistics and correlations between key variables.

**Variable**	***M***	***SD***	**1**	**2**	**3**	**4**	**5**	**6**
1. COVID-19 related stress	26.83	10.43	–					
2. Anxiety	4.70	3.46	0.609^***^	–				
3. PTG	48.54	21.24	0.213^***^	0.070	–			
4. Depression	12.91	9.90	0.497^***^	0.738^***^	−0.024	–		
5. Social support	62.39	11.09	−0.175^***^	−0.384^***^	0.355^***^	−0.482^***^	–	
6. Resilience	26.36	5.84	−0.161^***^	−0.297^***^	0.395^***^	−0.386^***^	0.494^***^	–

*** *P <0.001*.

### Structural Model and Mediation Analysis

**Figure 3:**
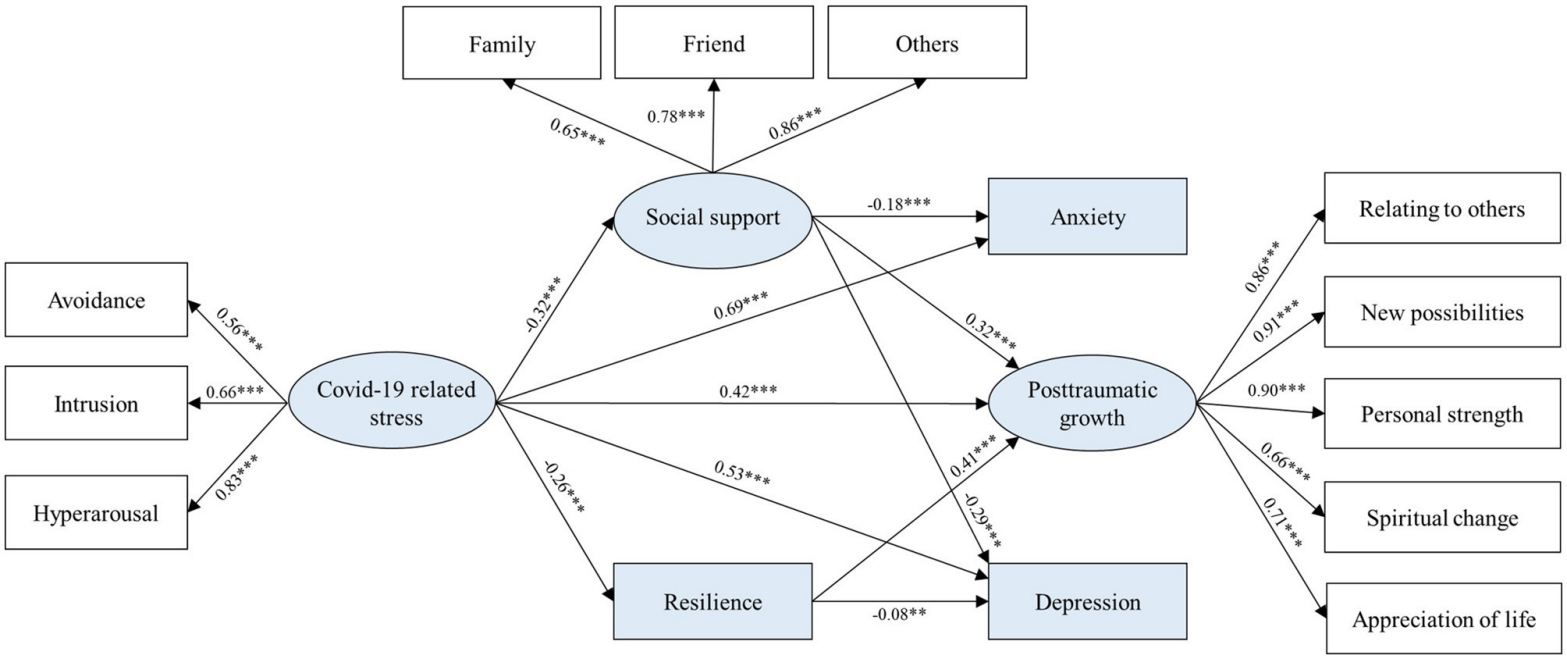
The mediating chain effect of social support and resilience in the relationship between the COVID-19 related stress and mental health outcomes (depression, anxiety, and posttraumatic growth). Sex, whether in only-child family, age, education, household income, and career were included as covariates. Standardized coefficients are reported. **P < 0.01, ***P < 0.001.

The SEM was used to explore the direct effect of the COVID-19 related stress on anxiety, PTG, and depression, as well as mediating pathways involving social support and resilience. At the same time, the COVID-19 related stress includes three latent variables: intrusion, avoidance, and hyperarousal; PTG includes five latent variables: relating to others, new possibilities, personal strength, spiritual change, and appreciation of life; social support includes three latent variables: family, friend, and others. The initial model analysis found the pathway that resilience mediated the relationship between the COVID-19 related stress and anxiety was not significant (*b* = 0.01, *P* = 0.281), therefore, this pathway did not enter the final model (Figure 3). The following information is about the final model. The fit indices indicated a good model fit, χ2 = 619.90, *df* = 144, *P* < 0.001, RMSEA = 0.07 with 90% CI [0.060, 0.071], CFI = 0.92, TLI = 0.90. The conceptual model mentioned in the introduction was confirmed, except only one mediation pathway was not significant after controlling demographics variables. As shown in the final model, the COVID-19 related stress positively predicted anxiety, PTG, and depression. The higher COVID-19 related stress was related to the lower social support, and further related to the higher anxiety, and the higher depression. The same results were achieved when resilience was the mediator, except the resilience to anxiety pathway was not significant. However, when it came to the PTG, the direct and indirect effects were inconsistent when social support and resilience were mediators in the relationship between the COVID-19 related stress and PTG, which indicated that both mediators were suppressed mediators. Specifically, the higher COVID-19 related stress predicted lower social support and resilience, resulting in a lower PTG. The direct and indirect effects with 95% CI of all mediation pathways are presented in Table 3.

**Table 3:** Direct pathway and indirect pathway coefficients of the final model.

		***b***	***S.E*.**	**95% CI**	***P***
COVID-19 related stress - > Anxiety	Direct pathway	0.689	0.030	[0.626, 0.743]	< 0.001
	Indirect pathway (mediated by social support)	0.058	0.014	[0.035, 0.091]	< 0.001
COVID-19 related stress - > PTG	Direct pathway	0.421	0.040	[0.343, 0.499]	< 0.001
	Indirect pathway (mediated by social support)	−0.101	0.024	[−0.155, −0.061]	< 0.001
	Indirect pathway (mediated by resilience)	−0.108	0.023	[−0.158, −0.068]	< 0.001
COVID-19 related stress - > Depression	Direct pathway	0.526	0.039	[0.447, 0.601]	< 0.001
	Indirect pathway (mediated by social support)	0.093	0.019	[0.060, 0.136]	< 0.001
	Indirect pathway (mediated by resilience)	0.021	0.009	[0.007, 0.041]	0.015

## Discussion

Due to effective COVID-19 prevention measures, China was the first country to experience a great fall of coronavirus cases and achieved control of the pandemic during last year (49). Nevertheless, the COVID-19 pandemic has impacted mental health and developed COVID-19 related mental problems among Chinese people (50, 51). The present study supported three of our hypotheses, which were (a) COVID-19 related stress was positively associated with depression, anxiety, and post-traumatic growth; (b) resilience and social support were negatively associated with depression and anxiety but positively associated with post-traumatic growth; (c) resilience and social support mediated the relationship between COVID-19 related stress and mental health outcomes (depression, anxiety, and post-traumatic growth). Consistent with our first hypothesis, COVID-19 related stress positively predicted depressive and anxiety symptoms. These results are in line with the previous studies that indicated individuals who perceived more stress about an event were more vulnerable to developing further mental problems (6, 52).

Meanwhile, Chinese people reported post-traumatic growth one year after the peak of the COVID-19 outbreak in the current study. Our results confirmed that individuals could learn and recover through adversity, which referred to post-traumatic growth reported in previous literature (12, 53). The current study also demonstrated that the COVID-19 related stress promoted Chinese people to develop post-traumatic growth. This result integrates well with the previous findings that indicate psychological distress and growth coexisted after facing adversity (54–56). Interestingly, post-traumatic growth was not correlated with depression and anxiety symptoms, indicating that the two types of changes were independent. One year after the peak of the COVID-19 pandemic, Chinese people developed both positive and negative changes after experiencing the COVID-19 related stress.

The current results also support our hypothesis 2, resilience and social support were negatively correlated with COVID-19 related stress, depression, anxiety and positively correlated with posttraumatic growth. As shown in previous studies, we confirmed the well-established negative link between resilience and psychosocial factors. For instance, Afshari et al. investigated resilience among nurses from hospitals and identified that the increase in stress was associated with a lower level of resilience during the COVID-19 pandemic (57). Another study also demonstrated the negative association between resilience and psychosocial problems, including depression and anxiety (58). Thus, understanding the importance of these psychological factors help with the improvement of Chinese's resilience, especially when being involved in a stressful environment arising from pandemic. Similarly, social support was negatively associated with stress, depression, and anxiety symptoms in China (59). Hence, public health management is encouraged to facilitate policies that include training in resilience and supplying social support to attenuate the negative mental impact of the COVID-19 pandemic and achieve post-traumatic growth.

Furthermore, our study confirmed hypothesis 3, resilience and social support played indirect roles in the association between COVID-19 related stress and negative as well as positive mental health outcomes. Resilience and social support are essential sources to facilitate mental well-being and improve people's understandings of meaning in life (17, 26). Recent studies reported that people might not seek help to relieve themselves due to the low resilience and perceived social support under the stress of the COVID-19 pandemic, which in turn results in new mental health problems (3, 4). In the case of the current study, COVID-19 related stress exacerbated the prevalence of depressive and anxiety symptoms by decreasing the Chinese people's perceived social support. The association between COVID-19 related stress and depressive symptoms was similarly mediated by resilience, but not anxiety symptoms. The results suggest that social support has a more significant role than resilience in explaining the impacts of COVID-19 related stress on anxiety symptoms. The restrictive measures on social distancing and quarantine in China may account for the critical role of social support in Chinese mental health during the pandemic, as social support resources may not be available when needed (60).

The decrease in resilience and perceived social support had a negative effect on post-traumatic growth as well. For one thing, COVID-19 related stress directly and positively predicted post-traumatic growth. As the previous studies illustrated, people gain post-traumatic growth from trauma or difficult conditions (13). For another, resilience and social support suppressed the prediction of COVID-19 related stress on post-traumatic growth. Under the pandemic, people with higher perceived stress experienced less resilience and social support, consequently perceiving less post-traumatic growth. However, in the whole effect, COVID-19 related stress still facilitated the development of post-traumatic growth. The challenges of COVID-19 related stress led to positive changes in Chinese people's attitudes and values toward life (61). A semi-structured interview study evaluated Chinese people's experience of post-traumatic growth and implied that people had a desire to improve relationships with their family and friend (61). However, the current study suggested that a higher level of COVID-19 related stress decreased Chinese people's resilience and perceived social support. Thus, the PTG targeted training can consider as future interventions to increase social support and resilience, therefore, to recover from the pandemic-related psychological distress.

## Limitations

This study has some limitations. First, the findings on the COVID-19 related stress were examined by cross-sectional data. It is difficult to make causal inferences on the association without testing the long-term consequences of the COVID-19 pandemic. Researchers are encouraged to expand the findings by designing a longitudinal experiment. Second, in the study, we only selected the Chinese public as our participants, which is hard to be representative of the whole population. Future studies can also study the different populations, including COVID-19 survivors, to further explore the mental health consequences of the COVID-19 pandemic and provide new insights to the local community and mental health services (4). Finally, the response bias from participants is possible for the self-reported design. Overall, more research is needed to generalize the results in the current study by performing cautiously.

## Conclusions

In all, the current study expands the understanding of the positive and negative psychological impacts in the aftermath of the COVID-19 pandemic. Our findings suggested that COVID-19 related stress positively predicted depression, anxiety, and post-traumatic growth. Resilience and social support concurrently mediated the associations. In anticipation of an increase in COVID-19 related stress in other countries, interventions are needed to address the emergent challenges in the future. For instance, mental health services could be prepared to screen and identify mental health issues, as a result, to provide proper treatments. Moreover, public health policies and strategies encouraged to design to facilitate resilience and social support (e.g., helping people connect during isolation or telepsychiatry) adapted to COVID-19 specific needs (62).

## Data Availability Statement

The generated datasets for this study can be found in the OSF (https://doi.org/10.17605/OSF.IO/5Z36P).

## Ethics Statement

The studies involving human participants were reviewed and approved by Institutional Review Board of Kangning Hospital. The patients/participants provided their written informed consent to participate in this study.

## Author Contributions

JH, YH, and JW: designed the study. YZ, XX, and JL: participated in the data collection. YH: analyzed the data. JH: advised on methodology. JH, YH, and JL: drafted the manuscript. JH, YZ, and JW: edited the manuscript and supervised data collection. All authors contributed to the article and approved the final manuscript.

## Funding

This study was supported by the Shenzhen Science and Technology Research and Development Fund for Sustainable Development Project (No. KCXFZ20201221173613036) and Shenzhen Key Medical Discipline Construction Fund (No. SZXK041).

## Conflict of Interest

The authors declare that the research was conducted in the absence of any commercial or financial relationships that could be construed as a potential conflict of interest.

## Publisher's Note

All claims expressed in this article are solely those of the authors and do not necessarily represent those of their affiliated organizations, or those of the publisher, the editors and the reviewers. Any product that may be evaluated in this article, or claim that may be made by its manufacturer, is not guaranteed or endorsed by the publisher.
