# Supplementary material for: COVID-19 Related Stress and Mental Health Outcomes 1 Year After the Peak of the Pandemic Outbreak in China: the Mediating Effect of Resilience and Social Support
Source: Front Psychiatry. 2022 Feb 21;13:828379. doi: 10.3389/fpsyt.2022.828379 (PMC8898823; doi:10.3389/fpsyt.2022.828379)
Supplement: Supplementary file 1 [file Table_1.DOCX]

Supplementary Material

# Supplementary Tables

**Supplemental Table 1**. *Impact of Events Scale-Revised (IES-R) for COVID-19.*

| Instructions:  Below is a list of items that describe. For each item, please rate how distressing each difficulty has been for you DURING THE PAST SEVEN DAYS with respect to COVID-19. | 0 | 1 | 2 | 3 | 4 |
| --- | --- | --- | --- | --- | --- |
| 1. Any reminder brought back feelings about COVID-19. |  |  |  |  |  |
| 1. I had trouble staying asleep. |  |  |  |  |  |
| 1. Other things kept making me think about COVID-19. |  |  |  |  |  |
| 1. I felt irritable and angry. |  |  |  |  |  |
| 1. I avoided letting myself get upset when I thought about it or was reminded of COVID-19. |  |  |  |  |  |
| 1. I thought about COVID-19 when I didn't mean to. |  |  |  |  |  |
| 1. I felt as if COVID-19 hadn't happened or wasn't real. |  |  |  |  |  |
| 1. I stayed away from reminders of COVID-19. |  |  |  |  |  |
| 1. Pictures about COVID-19 popped into my mind. |  |  |  |  |  |
| 1. I was jumpy and easily startled. |  |  |  |  |  |
| 1. I tried not to think about COVID-19. |  |  |  |  |  |
| 1. I was aware that I still had a lot of feelings about COVID-19, but I didn't deal with them. |  |  |  |  |  |
| 1. My feelings about COVID-19 were kind of numb. |  |  |  |  |  |
| 1. I found myself acting or feeling like I was back at that time. |  |  |  |  |  |
| 1. I had trouble falling asleep. |  |  |  |  |  |
| 1. I had waves of strong feelings about COVID-19. |  |  |  |  |  |
| 1. I tried to remove COVID-19 from my memory. |  |  |  |  |  |
| 1. I had trouble concentrating. |  |  |  |  |  |
| 1. Reminders of COVID-19 caused me to have physical reactions, such as sweating, trouble breathing, nausea, or a pounding heart. |  |  |  |  |  |
| 1. I had dreams about COVID-19. |  |  |  |  |  |
| 1. I felt watchful and on-guard. |  |  |  |  |  |
| 1. I tried not to talk about COVID-19. |  |  |  |  |  |

*Note.* This scale offers 5 different answer options which include options: Not at all, A little bit, Moderately, Quite a bit, Extremely.
